# Job Stress and Working Capacity among Fly-In-Fly-Out Workers in the Oil and Gas Extraction Industries in the Arctic

**DOI:** 10.3390/ijerph17217759

**Published:** 2020-10-23

**Authors:** Yana Korneeva, Natalia Simonova

**Affiliations:** 1 Department of Psychology, Northern (Arctic) Federal University named after M.V. Lomonosov, Arkhangelsk 163002, Russia; 2 Laboratory of Labor Psychology of the Faculty of Psychology, Moscow State University named after M.V. Lomonosov, Moscow 125009, Russia; n23117@mail.ru

**Keywords:** job stress, working capacity, fly-in-fly-out work, cortisol, objective, subjective (perceived) and projective stress parameters, oil and gas extraction industry, industrial psychology

## Abstract

(1) Background: the research purpose is to identify and describe the stress and working capacity dynamics of oil and gas fly-in-fly-out (FIFO) workers in the Arctic during the fly-in period using biochemical, psychophysiological and psychological methods with further analysis of the relationship between them using objective, subjective and projective indicators. (2) Methods: The research involved 70 oil and gas FIFO specialists in the Arctic. The study of stress and working capacity was carried out using biochemical (saliva analysis for cortisol), psychophysiological (complex visual–motor reaction and variational cardiointervalometry) and psychological (questionnaire “Well-being. Activity. Mood”, M. Luscher’s color test and the subjective control level methods. (3) Results: There is a similarity in the dynamic curves of oil and gas FIFO employees’ stress and working objective, subjective and projective indicators during the fly-in period. The maximum relationships number was obtained between objective cortisol indicators in saliva (stress), complex visual–motor response indicators (operator working capacity), variational cardiointervalometry (functionality level), and interpretation coefficients (working capacity, stress, vegetative balance) according to M. Luscher’s test. (4) Conclusions: The obtained results made it possible to explain the mechanisms underlying the previously developed FIFO workers’ adaptation strategies classification, in which emergency and economic adaptation strategies were identified.

## 1. Introduction

As demand for resources continues to grow around the world, an increasing number of mining projects are located in remote regions. Due to the remote location of natural resources, oil and gas companies have introduced a special method of labor organization for their employees—a fly-in-fly-out method or FIFO [1]. A fly-in-fly-out basis (FIFO) is defined as “any employment in which work is so isolated from workers’ homes that they are provided with food and housing in the workplace, and schedules are created whereby workers spend a fixed number of days at the field followed by a fixed number of days at home” [2]. One of the key industries using a fly-in-fly-out work organization is oil and gas production. According to research data, the leading harmful and hazardous factors in oil production and oil refining industries are harmful substances that periodically exceed the maximum permissible concentration by 3–4 times, as well as noise exceeding the maximum permissible level, and significant physical and neuro-emotional stress [3,4,5,6,7,8]. This indicates that work in oil and gas production is carried out in extreme conditions.

Fly-in-fly-out work at oil and gas production facilities imposes special requirements on professional adaptation of workers, which is due to the combination of many adverse environmental factors and the unpredictability of stressful or emergency situations [9,10,11,12,13,14].

Adaptation is always unfinished in such conditions and the adaptation process will result in newly-formed adaptation strategies. In our previous studies the classification of adaptation strategies (emergency or economical; stereotyped or differentiated) was developed and the prevailing types of adaptation strategies for fly-in-fly-out workers in the Far North and the Arctic were identified [15,16].

The peculiarity of northern climatic conditions, the intensity of physical activity inherent in FIFO work, group isolation, information deficiency and fatigue impose increased demands on the activity of all functional systems of the body [17]. In this connection, our earlier studies were carried out at various industries using FIFO: onshore oil and gas production [7,15,16,18,19], offshore oil and gas [20], diamond [15,16,20], logging [15,16], construction of main gas pipelines [8,20] and large industrial facilities—bridges [20]. These industrial facilities were located in different climatic zones (North, Arctic, South), assumed a different group isolation degree and the fly-in periods duration varied from 14 to 52 days.

The consequence of the complex influence of the factors on fly-in-fly-out personal is the development of workers’ unfavorable functional states, a decrease in their health levels, a decrease in the duration of active life and the onset of occupational diseases [5,6,7,8,14,17,18,21,22,23,24]. In our earlier works we studied the states dynamics (stress, working capacity, etc.) of fly-in-fly-out workers from various industries during the entire fly-in period with different fly-in duration (14, 28 or 52 days) [25,26,27,28]. It has been established that the dynamics of workers’ functional states’ is determined by the fly-in period stages: regardless of the fly-in duration (14, 28 or 52 days), in the workers’ state similar changes are observed at the beginning, middle or end of the fly-in period. In our opinion, this testifies to the influence of time perception on the fly-in period and the fly-in period image in workers’ minds, as well as to the special regulation and adaptation mechanisms that control these changes [26,29].

Bowers, Lo, Miller, Mawren and Jones studied the prevalence and correlation of psychological disorders in FIFO workers in the mining and construction industry in Australia. It was found that 311 respondents (28%) had high or very high levels of psychological stress, compared with 10.8%—typical for Australian residents in general. The most frequently reported stressors are: lack of special events (86%), problems in relationships with partners (68%), financial stress (62%), shift schedules (62%) and social isolation (60%). High psychological stress was significantly more likely in workers aged 25–34 and employees working a two-week schedule after one week of rest [30].

In studies [31,32,33,34], the stress level and the mood dynamics of employees and partners during the fly-in and fly-out periods, and, in particular, during the transition periods are determined. They show that there are differences in physiological and perceived stresses of FIFO workers.

A number of studies have established changes in stress levels and moods of employees and their partners during fly-in and fly-out periods, and, in particular, during transition periods, which were characterized by the study participants as stress, fatigue and disagreement periods [31,32,33,34,35,36].

Other studies have found no relationship between perceived stress levels (based on self-reported well-being) and physiological stress levels [37,38,39,40,41,42,43]. However, different perceived stress forms have different effects on the physiological stress level. Stress experiences associated with uncertainty, novelty, distress, anxiety, helplessness or lack of control are more important in triggering responses to physiological stress than other types of stress (e.g., habitual or routine stress) [41,44].

Some studies have shown a significant relationship between perceived and physiological stress levels [45,46,47].

This study was carried out in continuation of the work of the above authors. The uniqueness of our study is due to the expansion of the studied states (stress and performance), as well as the range of diagnostic techniques.

Biochemical, psychophysiological and psychological methods and techniques for diagnosing functional states are used in scientific research. In this connection, we decided to carry out a comparative analysis of the workers’ functional states dynamics in oil and gas production in the Arctic, using various methods. As a result of comparing the data obtained, we will be able to assess the specificity and versatility of the diagnostic techniques used in the context of reducing for both the subjects and the researchers.

The research purpose is to identify and describe the work stress and working capacity dynamics of oil and gas fly-in-fly-out workers in the Arctic during the fly-in period using biochemical, psychophysiological and psychological methods with further analysis of the relationship between objective, subjective and projective indicators.

Research question: to understand how objective, subjective and projective indicators of oil and gas employees’ stress and working capacity are related during a fly-in period.

Hypothesis: based on the available information about repeatedly recorded correlation of various indicators of functional states with the indicators of workers’ personal locus of control in our empirical studies [25,26,27,28] and unproductive neuropsychic stress as a predictor of professional deformations and professional maladjustment development; we assume the presence of the mechanisms of dynamic shifts, objectively and subjectively manifested stress and working capacity, mediated by the use of emergency and economical adaptation strategies.

Research stages and tasks:

1. Evaluate the similarity and difference between the dynamic curves of objective, subjective and projective indicators of stress and working capacity among oil and gas employees during a fly-in period.
2. Analyze the relationship of shifts in objective, subjective and projective indicators of stress and working capacity among oil and gas workers during a fly-in period in the context of relationships with the locus of control in different areas and the general level of unproductive neuropsychic stress.
3. Identify and describe the relationship of unproductive neuropsychic stress as a predictor of occupational deformations and professional maladjustment with FIFO work experience.

## 2. Materials and Methods

### Research Methods

1. The biochemical ones involve examination of subjects for cortisol concentration in saliva.

Cortisol in saliva is a direct indicator reflecting the concentration of free (biologically active cortisol) in blood, since the cell membrane (barrier) of the salivary glands does not allow biological molecules with a mass of > 400 Da to enter salivary ducts [48,49]. The free cortisol form, which has a small mass, freely penetrates into the salivary duct, while its protein-bound form is in blood and cannot pass through this barrier. The cortisol level in saliva does not depend on the volume and rate of its secretion, which is of fundamental importance in interpreting the results [50,51].

Under normal conditions, the cortisol rate changes depending on the time of day, namely: it rises in the morning, and gradually decreases in the evening; therefore, saliva was collected from workers during the entire fly-in period at regular intervals.

The material for the study was the saliva obtained from the subjects. Since the free cortisol concentration in saliva obeys a daily rhythm (in the daytime this indicator decreases by 54%, and in the evening hours—by 89%), the subjects’ saliva was collected in a special container from 5.00 to 5.30 in the morning.

The prerequisites were the exclusion of smoking, drinking, eating, chewing gum, and also not brushing your teeth 30 min before the examination.

Before the study, the samples were frozen and stored at a temperature of minus 20 °C. Before the analysis, the samples were thawed at room temperature, centrifuged for 10 min at 2000–3000× *g* in order to separate the shaped elements. The norm of the cortisol content in saliva < 19.1 nmol/L.

2. Psychophysiological methods involved using the device for psychophysiological testing—UPFT-1/30 “Psychophysiologist” (MTD Medikom, Taganrog, Russia):

a. Complex visual–motor reaction (CVMR)—the time of a complex visual–motor reaction (reaction with switching), characterizes the speed of excitation along the reflex arc.

The essence of the CVMR technique is to determine the time and stability of the visual–motor reaction to light stimuli (green and red squares in the center of the monitor screen). The statistical indicators analysis of the time a complex visual–motor reaction makes it possible to evaluate, in addition to the absolute time of the reaction, stability, the probability of errors and disruptions. The characteristics of the reaction time distribution make it possible to assess the stress degree, personal readiness to work, the fatigue degree and in some cases, the presence of pathological functional disorders or organic disorders of the central nervous system.

It allows the operator to assess the operator working capacity level on two alternative parameters of a complex visual–motor reaction (75 stimuli).

b. Variational cardiointervalometry (VCM) is the assessment of the functional state and adaptive capabilities of the cardiovascular system by the variational cardiointervalometry method. The duration is 128 cardiocycles and 5 min. It allows us to identify workers’ condition, characterized by overstrain and depletion of regulatory mechanisms.

The functional capabilities level (FCL)—this criterion is the most complete one and allows one to judge the ability to form a functional system adequate to a task and keep it for a long time [52].

3. Psychological methods:

c. The color preference test (M. Lusher, Russian adaptation by L.N.Sobchik) [53,54,55,56,57,58,59]. This is aimed at identifying the emotional and characterological basis of a person and their current state. To use the data from M. Luscher’s test, the interpretation coefficients developed by G.A. Aminev [60] were employed. On the factor analysis basis, he identified the following coefficients: heteronomy, concentricity, balance of personality traits, balance of the autonomic nervous system, working capacity and stress state. All these coefficients are calculated according to the appropriate formulas reflecting a particular combination of colors (Table A1).

d. The self-assessment questionnaire of states “Well-being. Activity. Mood” (WAM), developed by V.A. Doskin, N.A. Lavrentyeva, V.B. Sharay and M.P. Miroshnikov in 1973 [61]. WAM is a table that contains 30 pairs of words reflecting the studied features of psychoemotional state (well-being, mood, activity), expressed in polar ratings.

The construct validity of the WAM was established on the basis of comparison with the results of psychophysiological methods, taking into account the indicators of the flickering critical frequency, the temperature dynamics and chronoreflexometry of the body. The current validity was established by comparing the data of the contrast groups and the results of the subjects at different times of the working day [61].

Well-being is a set of subjective sensations that reflect a degree of physiological and psychological comfort of a person’s state, the direction of thoughts, feelings, etc.

Activity is one of the temperament manifestation areas, which is determined by the intensity and volume of a person’s interaction with physical and social environment. According to this parameter, a person can be inert, passive, calm, proactive, active or impetuous.

Mood is a relatively long-term, stable state of a person, which can be represented as (1) an emotional background (uplifted, depressed) i.e., an emotional reaction not to the direct consequences of specific events, but to their significance for the subject in the context of general life plans, interests and expectations; (2) a clearly identifiable state (boredom, sadness, melancholy, fear, enthusiasm, joy, delight, etc.).

Mood, in contrast to feelings, is always directed towards one or another object. Mood, being caused by a specific reason, manifests itself in the features of a person’s emotional response to influences of any nature.

It should be mentioned that when analyzing the functional state, not only the values of its individual indicators are important, but also their ratio. The fact is that a rested person’s assessments of activity, mood and well-being are usually approximately equal. As fatigue grows, the ratio between them changes due to the relative decrease in well-being and activity compared to mood.

e. Questionnaire—“the subjective control level” (SCL), J. Rotter (locus of control scale), in the adaptation of E.F. Bazhin, E. Golynkina, L.M. Etkind [62]. This technique is based on the locus of control concept by J. Rotter [63,64,65].

Locus of control is a psychological factor that characterizes a particular type of personality. It represents a person’s tendency to attribute responsibility for events occurring in life and the results of their activities to external forces (external locus of control) or their own abilities and efforts (internal locus of control).

People with an external locus of control, inclined to explain the consequences of their actions by the influence of circumstances, are usually called externalities, since they attribute responsibility for their activities exclusively to external conditions. The opposite type is internals. People of this type consider only themselves responsible for the results of their actions. Even if the circumstances are unfavorable, the internals will not make excuses for mistakes or failures.

However, Rotter considers the locus of control to be universal in relation to all types of situations: the locus of control is the same in the area of achievement and in the area of failure.

When developing the SCL questionnaire E.F. Bazhin, E.A. Golynkina and L.M. Etkind proceeded from the fact that sometimes not only the unidirectional combinations of the locus of control are possible in different types of situations. This position also has empirical evidence.

In this regard, the test developers proposed to distinguish subscales in the diagnostic technique for the locus of control: control in achievement situations, in failure situations, in industrial relations, non-personal and family relations, and in health.

In total, the SCL questionnaire consists of 44 items. In order to increase the reliability of results, the questionnaire is balanced according to the following parameters: in terms of internality–externality; by the sign of emotion; by the direction of attribution.

Table A2, shows the characteristics of each of the SCL questionnaire scales.

The analysis of available literature sources demonstrates the active use of various objective, subjective and projective methods in different combinations to assess functional states. Obviously, the measurement of the same objects by different methods can give different results, and in the case of functional states, reflect their different levels.

Multivariate assessment of functional states is very attractive, but very time consuming. We consider it useful to obtain information about the general and different in one-step functional states measurement under the influence of extreme environmental factors by objective, subjective and projective methods.

In our research, we used the most common methods from these groups.

An objective stress measure is calculated by collecting saliva for cortisol. The objective working capacity indicator was calculated by instrumental psychophysiological diagnostics, “complex visual–motor reaction”.

The projective stress and working capacity indicators were measured using M. Lusher’s color test and the corresponding interpretation coefficients were calculated according to G.A. Amenev.

The subjective stress and working capacity indicators were measured using the survey questionnaire “Well-being. Activity. Mood.”

At the first research stage, we correlated the data on the working capacity and stress dynamics of FIFO personnel, measured by means of objective, subjective and projective indicators, which corresponds to the first research task.

The question of the set of methods necessary and sufficient for assessing stress and working capacity in extreme activities is still under discussion. In this connection, it is useful to study their correlation for further effective research planning related to the study of workers’ state. This is the second research task.

Many researchers [4,66] show that the functional states level is associated with work experience and FIFO experience. Workers start each next fly-in period with lower status indicators. At the same time, dynamic curves are more related to factors of seasonal, psychological and work environment. In order to neutralize the influence of work experience in the statistical model, we used the calculation of individual shifts of indicators (increments) from measurement to measurement:

- Increment 1—the indicator value in the middle of the fly-in period minus the indicator value at the beginning of the fly-in period, divided by their sum and multiplied by 100%;
- Increment 2—the indicator value at the end of the fly-in period minus the indicator value in the middle of the fly-in period, divided by their sum and multiplied by 100%;
- Total increment—the indicator value at the end of the fly-in period minus the indicator value at the beginning of the fly-in-period, divided by their sum and multiplied by 100%.

Next, we calculated the increments for all indicators taken into account when building a relationship model between objective, projective and subjective parameters of FIFO workers’ stress and work capacity.

When assessing the influence of extreme environmental factors, the most significant attention is effectively paid to the nature of changes in the indicators of functional states, therefore, in the correlation analysis we included not the absolute values of the indicators of functional states, but their increments (shifts).

Extreme working conditions require mobilization of human resources. With their lack, it is difficult for employees to adapt. A decrease in resources is obviously associated with the presence of intrapersonal conflicts, and, hence, unproductive neuropsychic tension, stiffness, instability, fatigue, the predominance of negative and asthenic experiences. The above phenomena are reflected in the coefficient “standard deviation from the autologous normal (SD)” (see Table A1).

The regulation of human functional states is obviously carried out at different levels: from physiological to existential. At the physiological level, the predictors are the body capabilities in relation to environment tension. The locus of control can act as a marker of regulation of existential manifestations, which reflects subjective responsibility or ability to control what is happening around a person, and interfere or help him overcome the extreme nature of environmental impact. As noted earlier in the introduction, the choice of the method “the level of subjective control” is due to large significant correlations number of internality indicators with the states and personality characteristics in our earlier studies.

When solving the third research task, we proceeded from the fact that significant differences in seniority characteristics among workers with different unproductive neuropsychic stress levels can demonstrate the cumulative effect of the influence of FIFO work extreme factors on professional deformations and maladjustment development.

Statistical analysis was carried out using descriptive statistics and analysis of variance with repeated measures, univariate analysis of variance and correlation analysis using Pearson χ^2^. Processing was carried out using the SPSS 23.00 software package (IBM, Moscow, Russian Federation).

## 3. Results

The study involved 70 men—oil and gas employees on the Nenets Autonomous Okrug territory (fly-in duration 30 days; fly-out duration 30 days). The sample description of age and FIFO work experience in the North representations Table 1.

The education level of the participants was as follows: 9.6% with general secondary education, 50%—secondary specialized, 11.5%—incomplete higher education and 28.8%—higher professional education.

Participation in the study was voluntary (by a written voluntary informed consent). The sample included representatives of various professional groups: oil and gas production operators (16 people), boiler operators (12 people), drivers (12 people), engineers and technicians (14 people) and maintenance workers (16 people).

The study did not include employees working on the night shift. Night shift work is an additional extreme factor and requires a separate sample.

The study is a dynamic observation of stress and working capacity various parameters in the same people, who are isolated in a FIFO camp during 30 days, at various stages of a fly-in period: at its beginning, middle and end.

The observation of the dynamics of functional states parameters in 70 employees is sufficient for this sample to be representative in relation to the general population—FIFO oil and gas personnel working in the Far North.

The 59-sample size and above is sufficient for the Piroson’s correlation coefficients reliability of 0.40 and above with a statistical power of 0.90. Our sample was 70 people, so we analyzed 0.40 and above [67,68,69].

Thus, a sample of 70 people with triple measurements is representative and effective in relation to the final general population of rotational personnel in the oil and gas industry of the Russian Federation in the Far North.

**Figure 1:**
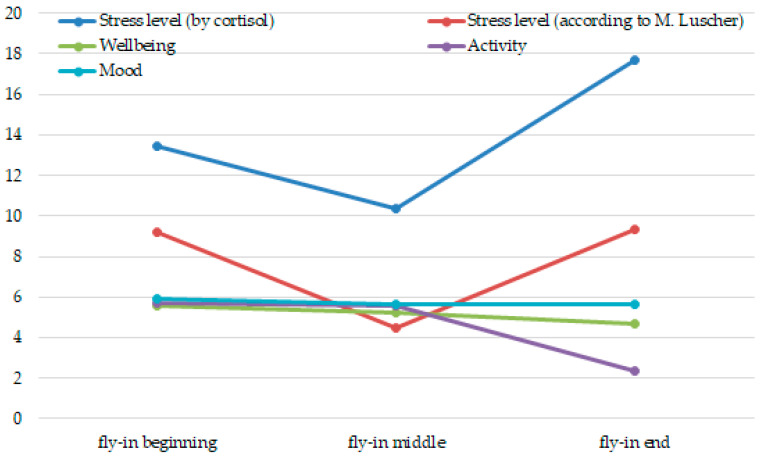
The stress dynamics among oil and gas employees during the fly-in period.

**Figure 2:**
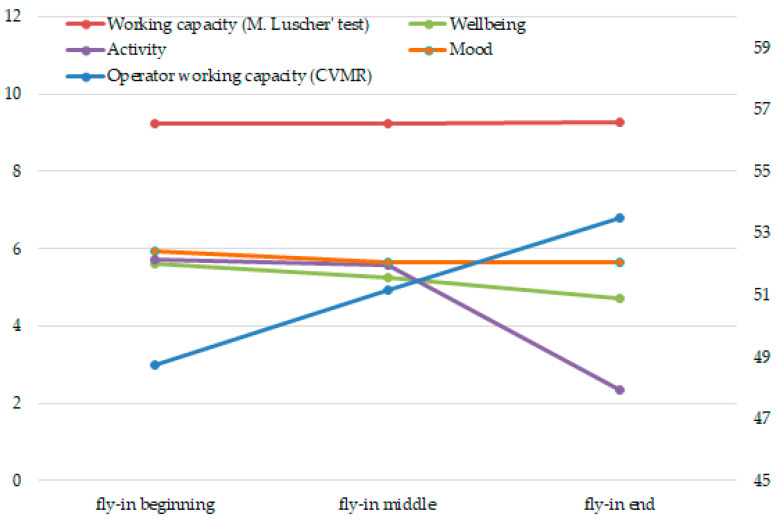
The working capacity dynamics among oil and gas employees during the fly-in period.

At the first research stage, we compiled the graphs of the working capacity and stress dynamics, measured by means of objective, subjective and projective indicators (Figure 1 and Figure 2).

The statistical significance of the differences in stress and working capacity indicators dynamics in the fly-in period was confirmed by the results of multivariate analysis of variance (ANOVA) with repeated measurements. We carried out seven ANOVA with repeated measurements for stress and working capacity indicators. The intragroup factor was three dimensions: the beginning, middle and end of the fly-in period. The significant value (sig., *p* ˂ 0.05) was achieved according to the Mauchly’s test of sphericity, therefore, to assess the result, we used the Pillai’s trace. The ANOVA results are presented in Table 2.

According to Figure 1, the stress level in terms of objective and projective indicators decreases by the fly-in period middle, which may be due to the completion of the work-in phase and the transition to the optimal functioning mode. Then, towards the end of the fly-in period, the stress level rises, which may be associated with a lack of internal resources and developing fatigue.

At the same time, according to subjective indicators, we observe the health state, activity and mood at the beginning and in the middle of the fly-in period among the personnel at a high level, by the end of the fly-in period the activity decreases. This is consistent with the data of objective and projective indicators.

According to the data in Figure 2, the employees’ efficiency increases from the beginning to the end of the fly-in period according to objective indicators. According to the projective indicator, it remains at the same level throughout the fly-in period, and, according to the subjective indicator, it decreases after the mid-fly-in period.

This research data is consistent with our earlier studies [26], when objective indicators (for example, an eye gauge, dynamometry) in this case of working capacity, which ensure the success of professional activity, remain unchanged, or slightly improve. Because they are controlled by the workers themselves, and this is a dominant feature, especially in the second half of the fly-in period, and ensure the success of activity.

Maintaining this success is associated with the depletion of resources and the search for physiological and psychological methods of maintaining performance. At the same time, the dynamics of psycho-physiological and psychological parameters have multidirectional tendencies, especially during the cross-sensitization period, when the human body and psyche are alternately trying to maintain the functional state at a high level.

At the next research stage, the objective, projective and subjective indicators of FIFO personnel’s stress and work capacity were correlated indirectly by the standard deviation from the autogenous norm (SD).

**Figure 3:**
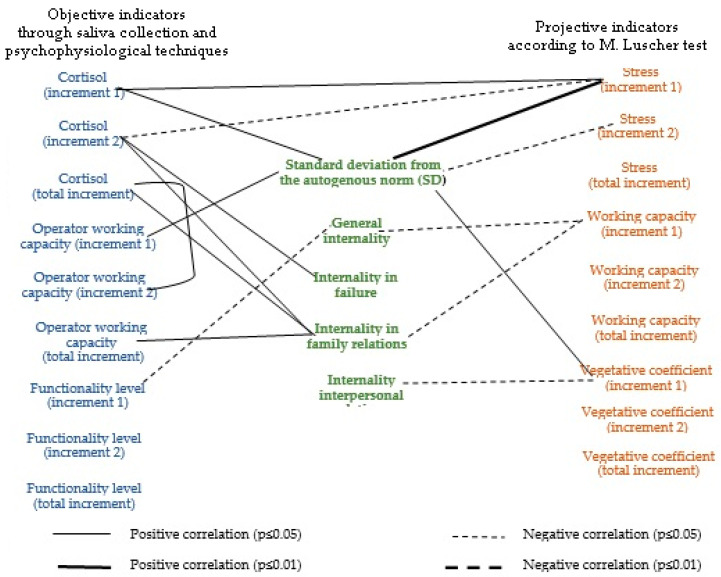
Correlation pleiad of relationship between objective and projective indicators of fly-in-fly-out (FIFO) workers’ stress and working capacity at an oil and gas production enterprise.

**Figure 4:**
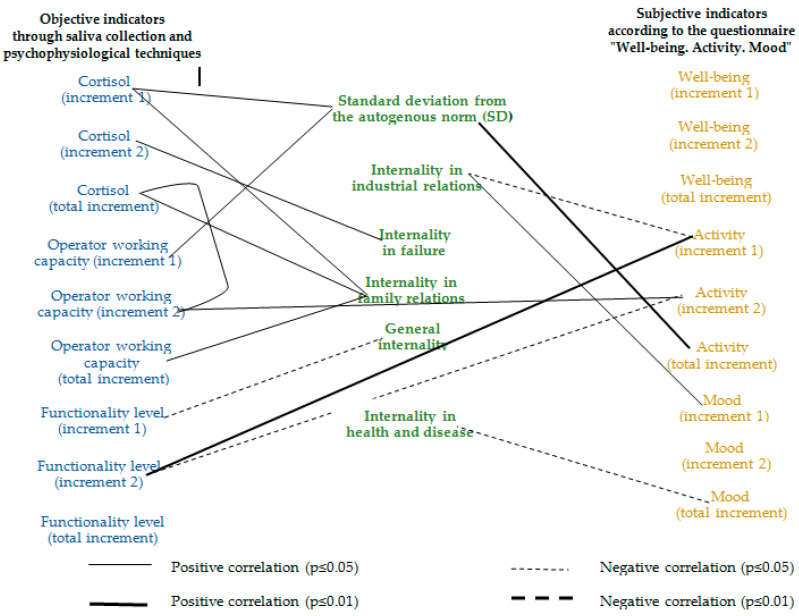
Correlation relationship pleiad between the objective and subjective indicators of FIFO workers’ stress and working capacity in oil and gas production enterprise.

**Figure 5:**
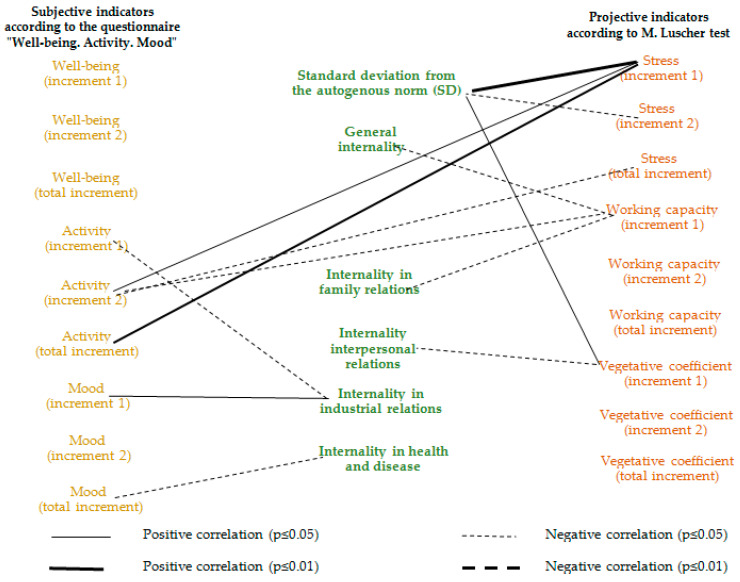
Correlation pleiad of relationship between subjective and projective indicators of FIFO workers’ stress and working capacity at an oil and gas production enterprise.

Correlation analysis was carried out using Pearson χ^2^ and three correlation groups were constructed: the relationship between objective and projective indicators (Figure 3), objective and subjective indicators (Figure 4) and projective and subjective indicators (Figure 5) of FIFO personnel’s stress and work capacity during the fly-in period.

As mentioned earlier, in order to be able to explain the adaptation and self-regulation mechanisms of workers’ states that occur during the fly-in period, we built pleiades indirectly by the total deviation from the autogenous norm and locus of control.

Table A1 demonstrates qualitative significant differences in connections relative to the first and the second shifts.

During the first half of the fly-in period, the key role, in our opinion, is played by the relationship between stress and the standard deviation from the autogenous norm, the level of cortisol, and the sympathetic part of the nervous system activation. All this ensures positive operator efficiency dynamics, i.e., good workability.

At the same time, the increase in working capacity according to M. Luscher’s test is in no way connected with objective indicators, but has a negative correlation with internality, especially in family relations. Such a projective self-assessment of working capacity is personal in nature.

The second half of the fly-in period dynamics is provided by the locus of control, especially in failures and family relations. The stress increase in the first half is inversely proportional to the cortisol increase in the second half of the fly-in period. The higher an employee’s responsibility for what is happening to his family, the more often he avoids choosing green, red and yellow colors, i.e., less workable.

Internality in family relations plays a key role in the relationships with the cortisol increase in general during the fly-in period and it’s the second half, in particular. Responsibility for failures is positively associated with cortisol increase in the second half of the fly-in period, which, in turn, is negatively associated with stress increase in the first half of the fly-in period.

Operator working capacity as an indicator of ensuring professional success in dynamic manifestations is, in general, positively associated with internality in the family relations. The operator’s working capacity increase in the second half of the fly-in period is provided by overall cortisol increase. The operator’s working capacity increase in the first half of the fly-in period is associated with the standard deviation from the autogenous norm.

Thus, unproductive neuropsychic stress in conjunction with the activation of the sympathetic division of the autonomic nervous system and an increase in discomfort with the choice of dark colors in the first position are associated with cortisol increase and provide adaptation to FIFO conditions, workability and the operator’s working capacity maintenance at the proper level.

In turn, the activation of the sympathetic division of the nervous system in the first half of the fly-in period is negatively associated with the locus of control in interpersonal relations. This phenomenon demonstrates the employee’s stress, due to group isolation and the perspective of forced communication with a limited group of people.

A high internality level is associated with cortisol increase in the second half of the fly-in period and over the entire period. This high level of increment in the second half of the fly-in period is negatively associated with stress according to M. Luscher’s test, i.e., the high level of tension in the first half of the fly-in period provides sufficient adaptation level and does not entail further cortisol increases.

At the same time, people with increased internality have higher cortisol levels in general and do not have a reserve for its adaptive jumps in the first half of the fly-in period. This explains the negative relationship between the stress increase in the first half of the fly-in period and the cortisol increase in the second.

Moreover, this hypothesis is supported by the relationship between the operator working capacity increase in the second half of the fly-in period with the total cortisol increase.

These results open up broad prospects for psychological support of FIFO personnel. We can work in the field of employee relations with family systems, and we can also adjust workability by training employees to self-regulate their states.

As can be seen from the data in Figure 4, the operator’s working capacity increase in the second half of the fly-in period is positively correlated with the increase in the workers’ activity self-assessment in the same period.

The activity increase in the first half of the fly-in period is positively associated with an increase in the workers’ functional capabilities level in the second half of the fly-in period, which indicates the resource preservation, based on the cardiovascular system state.

Based on the analysis of the second constellation, we can distinguish two different adaptation strategies to FIFO conditions, which correspond to the stayers and sprinters typology. High motivation to work and a cortisol adaptive burst in the first half of the fly-in period are associated with an increase in activity self-assessment and provide a cardiovascular system power increase in the second half of the fly-in period.

However, we see a negative relationship between this increase in the functional capabilities level with an activity increase in the second half of the fly-in period, i.e., having been actively involved in the work in the first half of the fly-in period, employees can afford to somewhat reduce their activity and calm down during its second half. This strategy corresponds to sprinters.

The same correlations indicate an increase in subjective activity and operator working capacity in the second half of the fly-in period due to the use of deep body resources in stayers.

Mood self-evaluation dynamics demonstrates the connection with the locus of control in the industrial relations and attitudes towards health. The rise in mood self-evaluation is expectedly associated with internality in industrial relations. Subjective acceptance of responsibility for what is happening at work corresponds to mood improvement at the stage of working in/activation (in the first half of the fly-in period).

The actively manifested internal locus of health control reduces the mood background in general during the fly-in period. An active interest in one’s own health obviously focuses a person’s attention on negative production factors of the environment and subjectively leads to a decrease in the mood background.

As can be seen from the data in Figure 5, changes in the workers’ activity subjective assessment from the middle to the end of the fly-in period are positively associated with stress and working capacity changes from the beginning to the middle of the fly-in period.

Those employees who had higher stress and working capacity indicators at the beginning of the fly-in period feel more active and demonstrate it in the second half of the fly-in period. As a result, they have lower stress indicators in the second half of the fly-in period (which demonstrates their negative relationship in the pleiad).

The choice of darker cards in M. Luscher’s test and the rejection of bright active colors is associated with unproductive neuropsychic tension and a subjectively evaluated activity increase in general during the fly-in period and especially in its second half.

We can assume that unproductive neuropsychic tension, stiffness, instability, fatigue, the predominance of negative and asthenic experiences is associated with an increase in projective stress indicators and subjectively assessed activity towards the end of the fly-in period and, ultimately, with the depletion of the workers’ body resources.

We assume that the relationship between the standard deviation from the autogenous norm and the sympathetic part of the nervous system activation in the first half of the fly-in period can play both a positive and a negative role in changing employees’ functional states.

In the case of physical and mental health, this beneficial sensitivity to environmental change ensures a healthy adaptive response at all levels (eustress).

In the case of mental and/or somatic ill-being, such activation becomes excessive and reduces functional capabilities and adaptability of a person. He manages to increase his activity only at the end of the fly-in period with an adverse mental reaction increase.

These connections demonstrate the manifestations of negative functional states and, if repeated, lead to personal deformities [70].

The results obtained made it possible to formulate the particular hypothesis that unproductive neuropsychic stress can have a cumulative effect and be associated with FIFO work experience. This was the third research task.

To test this hypothesis, we divided all the surveyed employees by the level of standard deviation from the autogenous norm severity into three groups: with low, medium and high levels of internal conflicts and unproductive neuropsychic stress (division based on the formula mean value ± 0.5 × standard deviation).

The division resulted in 29.0% of those surveyed having a low level, 27.4%—an average level, and 43.6%—a high level of unproductive neuropsychic stress.

Next, we conducted a univariate analysis of variance, where the dependent variable was the FIFO work experience, and the fixed factor was attribution to a group with a low, medium, or high standard deviation from the autogenous norm.

**Figure 6:**
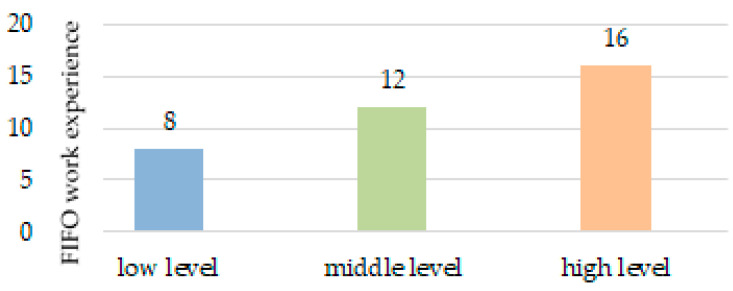
Oil and gas employees’ FIFO work experience with different levels of standard deviation from the autogenous norm.

According to the criteria for intergroup effects, there are statistically significant differences in the employees’ FIFO work experience with different levels of unproductive neuropsychic stress and internal conflicts (F = 4.455 at *p* = 0.016). Figure 6 provides a qualitative description of these differences.

As can be seen from Figure 6, our hypothesis that unproductive neuropsychic stress can have a cumulative effect and be associated with FIFO work experience was confirmed. This fact demonstrates the need for regular psychological work with personnel in reducing unproductive neuropsychic stress, etc.

Successful work of psychologists in this direction will contribute to an increase in the eustress share in relation to distress in workers’ functional states.

## 4. Discussion

The research results that in the first part of the oil and gas fly-in period, workers are in the working phase or activation (increased stress and neuropsychic tension), have numerous confirmations in the works of other researchers.

Clifford’s research has assessed whether employees’ stress levels and moods and their partners change during fly-in and fly-out periods and, in particular, during transition periods [34].

Transition periods have previously been defined as times of particular stress and the likelihood of misunderstandings and disputes [33,35,36].

Physiological stress levels (as measured by cortisol concentration in saliva on waking) varied throughout the fly-in and fly-out periods and were significantly higher during the transition period between going to work compared to periods during mid-shift and mid-holiday.

Similarly, there was a dynamic during the work beginning period, with significantly higher acute stress levels than those in the middle of a fly-in period or in the middle of a holiday.

In terms of stress specific sources during transition periods, almost all FIFO workers felt tired during the first one to two days of holidays, and up to a third of respondents reported that the first one to two days were stressful, requiring one to two days again to “fit” into the circle of loved ones at the beginning of the holidays and negative moods during the last one to two days off (according to the dissatisfaction scale).

These data are supported by studies of scientists from various countries, which reported on transition periods as periods of stress, fatigue and disagreement [31,32,33].

Numerous studies have also found that perceived stress levels (based on self-reported well-being) are independent of physiological stress levels [37,38,39,40]. This fact is also analyzed in this study using the example of the self-assessment questionnaire results analysis for well-being, activity and mood.

Hjortskov and colleagues suggest that normal perceived stress levels (based on self-reported well-being) do not elicit a physiological stress response, and a stress response is only triggered by perceived stress levels that are higher than normal [41]. In support of this assumption, there are numerous studies that showed that people with low perceived stress levels did not demonstrate a relationship between perceived and physiological stress levels [37,38].

Another factor to consider is that different forms of perceived stress have different effects on physiological stress levels. Stress experiences associated with uncertainty, novelty, distress, anxiety, helplessness, or lack of control are more important in triggering the physiological stress response than other types of stress (such as habitual or routine stress) [41,44].

Although cortisol concentrations are commonly used as a physiological stress biomarker [45], some studies have found a significant relationship between perceived and physiological stress levels [46,47], other studies have not found a reliable relationship between cortisol concentrations and stress levels based on self-reported subjects [37,38,39,40] or mood [42].

One previous study found a negative association between cortisol concentrations and perceived stress [43]. Clough et al. reviewed the studies that simultaneously measure perceived and physiological stress levels and were unable to suggest any plausible explanation why some studies found associations between the two and others did not, apart from the differences between the studies in methodology and externally controlled factors.

Our study recorded the relationship and similar dynamics of stress and working capacity indicators, measured using objective and projective methods. Conversely, no connections were found between the indicators of subjective and objective states. Based on this, it can be concluded that it is advisable to diagnose objective (using biochemical and psychophysiological methods) and projective psychological (using M. Luscher’s color test) parameters of FIFO workers’ stress and working capacity. The research program may not include subjective methods based on workers’ self-assessment of their states. This allows us to simplify the assessment procedure without reducing the effectiveness of the results obtained.

The data on the explanation of FIFO workers’ adaptation and self-regulation mechanisms of their states during the fly-in period have a high value in this study. These results made it possible to reveal and explain the previously developed classification of FIFO workers’ adaptation strategies, in which emergency and economical adaptation strategies were identified.

An emergency adaptation strategy is characterized by the fact that an employee from the very beginning of the fly-in period is actively involved in intensive work. Quickly spending his resources. This strategy type correlates with the sprinter’s strategy.

In our earlier studies [15,16], we assumed that this strategy type may be ineffective, since it involves the intensive expenditure of internal resources at the beginning of the fly-in period, and therefore, they will not be enough for the successful professional performance in the second part of the fly-in period.

However, this study shows that this is not the case, and an emergency strategy can be effective. The reason for such high activity in the first part of the fly-in period is the need to work through the stress that arises during the working-in phase (activation). Further, this is the key to the possibility of preserving, maintaining and slightly increasing working capacity in the second part of the fly-in period.

At the same time, we found that this adaptation strategy type is used, which is correct, by FIFO workers with a higher functional reserves level. Furthermore, that is why most of the FIFO workers in various industries use an emergency adaptation strategy type [20]. We can assume that work in extreme conditions of activity is chosen, as a rule, by people with a high functionality level. This gives them an advantage in resources to overcome the negative impact of the environment.

The economical adaptation strategy assumes the gradual expenditure of internal resources by employees during the fly-in period and correlates with the stayer type. As shown in this study, this strategy type is typical for workers with an average functionality level.

FIFO working conditions vary greatly at different enterprises and vary from weak to strong. Different tension levels trigger different mechanisms in the workers’ psyche and body, require different behavior strategies from him and, accordingly, lead to different consequences.

The analysis of stress indicator values dynamics revealed a tendency towards the development of the stress state in the middle of the fly-in period in all FIFO specialists. In accordance with our previous studies, it can be assumed that in the spring, when the study was conducted, the workers experienced the influence of physiological desynchronosis, that is, they became dependent on external factors, the impact of which leads a person into a stress state [16,29].

One of the possible directions for correcting employees’ unfavorable conditions during the fly-in period is the creation of the relaxation room, to help reduce the stress of workers who are engaged in hard work associated with increased concentration, physical, emotional and moral stress. It significantly reduces the risk of emergencies at enterprises whose work is related to operator activities, reduces the burden on employees whose professional duties are associated with negative emotions or whose work requires a particular concentration of attention. The relaxation room also helps motivate staff when choosing a place of work, provides contact with employees, helps to identify problems at workplaces, makes it possible to regulate job relationships and smooth out sharp corners, identifying problem areas in activities.

In the relaxation room, a complex method of mental processes optimization is used, conventionally called psychological relief, which includes physiological and psychological means of restoring working capacity and preventing overwork of emotional origin.

The main purpose of this room is to restore working capacity at the stage of developing fatigue.

The creation of such a room for FIFO workers in the Far North and the Arctic is intended for the active training of the skills of their mental states self-regulation in a wide range of life situations and for their rest in order to relieve fatigue and stress in a special place.

The limitations of this research are a fixed set of extreme climatic-geographical and industrial environmental factors that determine the intensity level of professional activity. We assume that in less severe climatic conditions, stress will be less pronounced and relationships may have a different nature.

The study included only day shift FIFO employees. This is due to a too small sample size of employees working on the night shift to provide statistically significant data.

Similarly, a small number of women working in this FIFO camp did not make it possible to study this issue with respect to women.

In the future, it would be interesting to continue our research according to the proposed experimental plan in different year seasons, in different climatic zones, with different number of workers living in FIFO camps (degree of group isolation).

## 5. Conclusions

The main research result is a revealed similarity in the dynamic curves of oil and gas FIFO employees’ stress and working objective, subjective and projective indicators during the fly-in period. The stress level in terms of objective and projective indicators decreases by the middle of the fly-in period, which may be due to the completion of the work-in phase (activation) and the transition to the optimal functioning mode. Then, towards the end of the fly-in period, the stress level rises, which may be associated with a lack of internal resources and developed fatigue.

Differences in the dynamics of the states were noted in terms of subjective well-being, activity and mood indicators: at the beginning and middle of the fly-in period they are at a high level, by the end of this period activity decreases.

The maximum number of connections was obtained between objective cortisol indicators in saliva (stress), complex visual–motor response indicators (operator working capacity), variational cardiointervalometry (functionality level), and interpretation coefficients (working capacity, stress, vegetative balance) according to M. Luscher’s test. The correlation between self-evaluation of well-being, activity and mood with objective and projective indicators are less significant and rather more trivial (the mood at the beginning of the fly-in period is higher among those who control industrial relations, a decrease in the mood background with high control over their health).

The dynamic shifts analysis in relation to the locus of control and unproductive neuropsychic stress revealed clear differences in the first and the second halves of the fly-in period. We have recorded a period of workability (the first half of the fly-in period) with an increase in levels in terms of cortisol, stress, operator efficiency and activity.

The results obtained made it possible to explain the mechanisms underlying the previously developed classification of FIFO workers’ adaptation strategies, in which emergency and economical adaptation strategies were identified. The emergency strategy type corresponds to the sprinter strategy, and the economical type corresponds to the stayer one. The data obtained indicate the effectiveness of both strategy types and their dependence on the internal energy potential of human resources.

An increase in the standard deviation from the autogenous norm was revealed with an increase in the employees’ FIFO work experience, which demonstrates an increase in maladjustment, which requires active intervention to maintain mental and physical health.

## Figures and Tables

**Table 1:** Sample characteristics.

Characteristics	Min–Max	Mean ± SD	25th Percentile	50th Percentile	75th Percentile
Age	24–60	38.46 ± 1.410	31.0	35.5	47.0
FIFO work experience in the North	0.5–31	9.53 ± 1.072	3.0	9.0	15.0

**Table 2:** The ANOVA results in relation to the stress and working capacity indicators.

Stress and Working Capacity Indicators	Unit	M ± SD in Beginning Fly-In	M ± SD in Middle Fly-In	M ± SD in End Fly-In	Pillai’s Trace	F	Hypothesis df	Error df	Sig. *p*
Stress level by cortisol content in saliva	nmol/L	13.4 ± 1.40	10.4 ± 1.57	17.7 ± 2.22	0.366	2.780	10.000	124.000	0.004
Stress level according to the M. Luscher’s method	points	8.2 ± 0.58	4.5 ± 0.64	9.4 ± 0.66	0.189	9.881b	2.000	85.000	0.000
Operator working capacity according to CVMR	points	47.7 ± 4.41	51.2 ± 4.91	53.5 ± 5.64	0.384	3.962	6.000	100.000	0.001
Working capacity according to M. Luscher’s method	points	9.2 ± 0.38	9.2 ± 0.30	9.3 ± 0.42	0.432	6.529	6.000	142.000	0.000
Well-being	points	5.6 ± 0.258	5.2 ± 0.37	4.7 ± 0.40	0.260	11.249b	2.000	64.000	0.000
Activity	points	5.7 ± 0.23	5.6 ± 0.27	2.4 ± 0.35	0.472	38.068b	2.000	85.000	0.000
Mood	points	5.9 ± 0.24	5.7 ± 0.34	5.7 ± 0.30	0.250	10.680b	2.000	64.000	0.000

## Appendix A

**Table A1:** Calculation and description of interpretation coefficients for M. Lusher’s test, developed by G.A. Aminev.

Interpretation Coefficients	Calculation *	High Values	Low Values
Heteronomy–autonomy	(blue spot + yellow spot) − (green spot + red spot)	Heteronomy (from 0 to plus 9.8) is passivity, a tendency to a dependent position on others, sensitivity	Autonomy (from 0 to minus 9.8) is independence, activity, initiative, independence, tendency to dominate, striving for success and self-affirmation
Concentricity–eccentricity	(blue spot + green spot) − (red spot + yellow spot)	Concentricity (from 0 to plus 9.8) is focusing on one’s own problems	Eccentricity (from 0 to minus 9.8) is interest in the environment as an object of influence or a source of assistance
Vegetative balance (VB)	(Red spot + yellow spot) − (Blue spot + black spot)	(From 0 to plus 9.8) is the predominance of the sympathetic nervous system, i.e., mobilization of all functions, preparation for active protection, flight	(From 0 to minus 9.8) is the predominance of the parasympathetic nervous system, i.e., the body’s work is aimed at rest, recuperation, saving resources
Balance of personality traits (PB)	(green spot + yellow spot) − (blue spot + red spot)	(0 to plus 9.8) is an unstable, contradictory personality	(From 0 to minus 9.8) is a balance of personality traits
Working capacity	green spot + red spot + yellow spot	(9.1 to 16) this is low working capacity	(from 16 to 20.9) this is high working capacity
Stress	C1 ** + C2 ***	If from 0 to 20, then there is a tendency to form stress	if more than 20, then this is a manifestation of a stressful state
Standard deviation from the autologous normal (SD) ****	To calculate the SD, it is necessary to compare the order of places that occupy the colors in the selection with their “ideal” location (red (3)—yellow (4)—green (2)—violet (5)—blue (1)—brown (6)—gray (0)—black (7)). First, the difference between the actual occupied space and the standard position of the color is calculated, and then these differences (their absolute values, without taking into account the sign) are summed up.	SD is an indicator of emotional discomfort degree, that is, from a state of absolute rest. The general meaning of the points can be determined as follows: 4 is statistical average sample rate; 3 and 5 is a slight deviation from the autogenous norm; 2 and 6 is significant deviation from the norm; 1 and 7 is an exceptionally large deviation from the norm. The higher the SD value, the more pronounced unproductive tension, tightness, instability, fatigue, the predominance of negative and asthenic experiences.	

Note: * M. Luscher identifies seven criteria, depending on where the given color stands. In turn, the position occupied by the color has a conditional score: 1st place—8.1 points; 2nd place—6.8 points; 3rd place—6.0 points; 4th place—5.3 points; 5th place—4.7 points; 6th place—4.0 points; 7th place—3.2 points; 8th place—1.9 points. ** C1 = 8.1 × x1 + 6.8 × x2 + 6 × x3, where x1, x2, x3 are the positions of the first three places, if at 1,2,3 positions are brown, gray, black, then one is substituted in the parenthesis. *** C2 = 6 × y1 + 6.8 × y2 + 8.1 × y3, где y1, y2, y3—the last places, if at 6,7,8 positions are blue, green, red, yellow, then the unit is substituted in brackets. **** After Wallneffer’ research [71], the 34251607 choice was adopted by M. Luscher as the norm of color preferences and is a reference indicator of neuropsychic well-being.

The color choice by the subject in the order of an autogenous norm presumably reveals an energetic, active subject who does not have any signs of fatigue, looking to the future with optimism, not burdened with deep personal problems and conflicts, confident, emotionally stable and balanced.

The lower the score of the subject’s SD, the closer he is to a similar standard of neuropsychic well-being. The higher the SD value, the more pronounced unproductive tension, tightness, instability, fatigue, the predominance of negative and asthenic experiences.

The numerical assessment procedure for the remoteness degree of the subject’s color preferences from the autogenous norm was proposed by A.I. Yurjev [72,73]. SD values range from 0 to 32 points and can only be even. Later, as the test psychodiagnostic capabilities were studied, a normalized statistical distribution of SD estimates was obtained, reduced to a standard seven-point scale later [74,75].

## Appendix B

**Table A2:** Characteristics of the “subjective control level” questionnaire scales.

Questionnaire Scales	Characteristic of a High Level of Expression	Characteristic of a Low Level of Expression
General internality scale	Reflects a high level of subjective control over any significant situations. Such people believe that most of the important events in their life are the result of their own actions. They can control them, and, thus, they feel their own responsibility for these events and for the way their life develops in general. The generalization of various experimental data allows us to speak of internals as more self-confident, calmer and more benevolent, more popular in comparison with the external ones. They are distinguished by a more positive system of attitudes towards the world and a greater awareness of the meaning and goals of life.	Corresponds to a low level of subjective control. Such people do not see the connection between their actions and the events of their lives that are significant to them, do not consider themselves capable of controlling their development. They believe that most of the events in their life are the result of an accident or actions of others. Generalization of various experimental data allows us to speak of externalities as people with increased anxiety and concern. They are distinguished by conformity, less tolerance towards others and increased aggressiveness, less popularity in comparison with internals.
Achievement internality scale	Such people believe that they themselves have achieved all the good that was and is in their life, and that they are able to successfully pursue their goals in the future.	Such a person attributes his success, achievements and joys to external circumstances—luck, good fortune, or the help of other people.
Failure internality scale	These traits are manifested in a tendency to blame oneself for various troubles and suffering.	Such a person is inclined to attribute responsibility for such events to other people or to consider them the result of bad luck.
Internality scale in family relationships	This person considers himself responsible for the events taking place in his family life.	This subject considers not himself, but his partners to be the cause of significant situations that arise in his family.
Internality scale in industrial relations	This person considers his actions to be an important factor in organizing his own production activities, developing relationships in the team, his promotion, etc.	A person tends to attribute more importance to external circumstances - leadership, workmates, luck or bad luck.
Internality scale in interpersonal relations	Such a person considers himself to be responsible for building interpersonal relationships with others.	A person is inclined to attribute more importance in this process to circumstances, an occasion, or to people around him.
Internship scale in health and disease	A person considers himself largely responsible for his health: if he is sick, then he blames himself for this and believes that recovery largely depends on his actions.	A person considers illness and health to be the result of an accident and hopes that recovery will come as a result of the actions of other people, primarily doctors.
